# 740 Implementation and Retrospective Assessment of a Modified Graft Loss Grading System

**DOI:** 10.1093/jbcr/irad045.215

**Published:** 2023-05-15

**Authors:** Adam Conner, Beverly Beaucock, Anjay Khandelwal, Mallory Wilson, Lauren Lewis, Richard Lou, Djoni Elkady

**Affiliations:** Akron Children's Hospital, Canton, Ohio; Akron Children's Hospital, Akron, Ohio; Akron Children's Hospital, Akron, Ohio; Akron Children’s Hospital, Akron, Ohio; Akron Children's Hospital, Akron, Ohio; Akron Children's Hospital, Akron, Ohio; Loyola University Stritch School of Medicine, Maywood, Illinois; Akron Children's Hospital, Canton, Ohio; Akron Children's Hospital, Akron, Ohio; Akron Children's Hospital, Akron, Ohio; Akron Children’s Hospital, Akron, Ohio; Akron Children's Hospital, Akron, Ohio; Akron Children's Hospital, Akron, Ohio; Loyola University Stritch School of Medicine, Maywood, Illinois; Akron Children's Hospital, Canton, Ohio; Akron Children's Hospital, Akron, Ohio; Akron Children's Hospital, Akron, Ohio; Akron Children’s Hospital, Akron, Ohio; Akron Children's Hospital, Akron, Ohio; Akron Children's Hospital, Akron, Ohio; Loyola University Stritch School of Medicine, Maywood, Illinois; Akron Children's Hospital, Canton, Ohio; Akron Children's Hospital, Akron, Ohio; Akron Children's Hospital, Akron, Ohio; Akron Children’s Hospital, Akron, Ohio; Akron Children's Hospital, Akron, Ohio; Akron Children's Hospital, Akron, Ohio; Loyola University Stritch School of Medicine, Maywood, Illinois; Akron Children's Hospital, Canton, Ohio; Akron Children's Hospital, Akron, Ohio; Akron Children's Hospital, Akron, Ohio; Akron Children’s Hospital, Akron, Ohio; Akron Children's Hospital, Akron, Ohio; Akron Children's Hospital, Akron, Ohio; Loyola University Stritch School of Medicine, Maywood, Illinois; Akron Children's Hospital, Canton, Ohio; Akron Children's Hospital, Akron, Ohio; Akron Children's Hospital, Akron, Ohio; Akron Children’s Hospital, Akron, Ohio; Akron Children's Hospital, Akron, Ohio; Akron Children's Hospital, Akron, Ohio; Loyola University Stritch School of Medicine, Maywood, Illinois; Akron Children's Hospital, Canton, Ohio; Akron Children's Hospital, Akron, Ohio; Akron Children's Hospital, Akron, Ohio; Akron Children’s Hospital, Akron, Ohio; Akron Children's Hospital, Akron, Ohio; Akron Children's Hospital, Akron, Ohio; Loyola University Stritch School of Medicine, Maywood, Illinois

## Abstract

**Introduction:**

Success of split-thickness skin grafting (STSG) is a cornerstone of modern burn care and sets the stage for success in acute, rehabilitative and reconstructive care. Shupp et al, proposed a graft loss grading system, which the authors then modified to reflect true clinical practice and changes in management. Purpose of the quality improvement (QI) project was to design, implement and assess this grading system.

**Methods:**

A modified graft loss grading system was developed and implemented. Burn patients requiring STSG were tracked over a five month period, and initial (first takedown) and subsequent (either outpatient or 7 days after initial assessment) graft loss grades and contributing factors were noted. Assessments were performed an APP and burn surgeon with agreement of an assigned grade. Patient demographic data was recorded.

**Results:**

63 patients underwent 66 STSG procedures during the review period (44 males, 19 females). Average age was 33.3 years (range: 1-86). Average % TBSA burn was 10.2% (range 0.2-67%). Obesity was present in 33.3% of patients, cardiac disease (hypertension, coronary artery disease or congestive heart failure) in 21.2%, DM in 21.2% and 24.2% of patients were active smokers. Flame burns represented the etiology in the majority (51.5%) of patients. Overall, 10 patients (16%) on initial assessment and 18 patients (29%) on subsequent assessment had evidence of graft loss. Etiology of graft loss included wound bed viability - the default etiology (10 patients/55%), infection (3 patients/17%) and hematoma/seroma (4 patients/22%). Of the 18 patients, 5 (28%) required re-grafting. Of patients with graft loss, 89% had a comorbidity including DM (28%) and smoking history (28%). Malnutrition was felt to contribute in 3 (17%) of patients.

**Conclusions:**

A graft loss grading system is a beneficial quality improvement project to characterize the surgical results of burn patients undergoing STSG. This modified grading system better characterizes graft loss outcomes and subsequent changes in management if applicable. Wound bed viability remains a major factor in graft loss, although the majority of these patients had a contributing comorbidity.

**Applicability of Research to Practice:**

Implementation of a graft loss grading system can be a valuable quality improvement tool that can be employed by burn centers to assessment surgical results.

